# Magnetic resonance urography in children – when and why?

**DOI:** 10.2478/v10019-011-0023-6

**Published:** 2011-07-20

**Authors:** Sandra Vegar-Zubovic, Spomenka Kristic, Lidija Lincender

**Affiliations:** 1 Clinic of Radiology, Clinical Centre of University of Sarajevo, Sarajevo, Bosnia and Herzegovina; 2 Academy of Sciences and Arts of Bosnia and Herzegovina, Sarajevo, Bosnia and Herzegovina

**Keywords:** MR urography, urinary tract, paediatrics

## Abstract

**Background:**

The aim of the study was to determine the potential of magnetic resonance urography (MRU) in evaluation of paediatric urinary tract pathologies.

**Patients and methods.:**

Twenty-one paediatric urological patients were evaluated with T1, T2 prior and after and 3D gradient echo sequences after the contrast administration. Results were compared with findings obtained with ultrasound which was performed to all of patients, intravenous urography performed to 14 patients with the diagnosis of hydronephrosis and voiding cystouretrography performed to 6 patients where hydronephrosis was suspected to be caused by vesicoureteral reflux (VUR).

**Results:**

MRU not only established the cause of hydronephrosis in all 14 cases (5 ureteropelvic junction (UPJ) stenosis, 1 functional stenosis, 3 residual hydronephrosis, 1 combination of UPJ and vesico-ureteric junction (VUJ) stenosis with hydromegaureter, 2 fetal ureters and 3 insufficient broad ureteral orifices), but gave additional information about existing pathological conditions in all of patients compared to other previously performed examination (1 caliceal lithiasis, 4 UPJ stenosis, 1 VUJ stenosis, 1 neurogenic bladder, 1 hypotonic ureter, 1 urinary infection, 1 duplication of pelvis and ureter, 1 urinary retention and 1 fetal ureter). Other MRU findings were: 3 polycystic kidney disease, 1 caliceal cyst, 2 simple renal cysts, 1 long hypotonic twisted ureters and 1 hypertrophied column of Bertini.

**Conclusions:**

Because of the ability to acquire high contrast and spatial resolution images of the whole urinary tract in any orthogonal plane, MRU enables a precise detection and differentiation of pathological urological conditions. We believe that in the future, because of its advantages, MRU will replace traditional methods in the evaluation of urinary tract pathologies.

## Introduction

Diagnostic imaging and therapy protocols of urological disorders in pediatric population are constantly changing because of the introduction and practical application of modern MRI and multi-slice CT (MSCT) modalities, use of fetal ultrasound, introduction of ultrasound as the obligatory part of the modern urodiagnostic protocol and cognition of the importance of urogenital pathology in children.

During the last seven years we have witnessed a significant development in the introduction of magnetic resonance urography (MRU) in the diagnostic protocol.^1^–^3^ MRU displays the combination of the internal spatial and contrast resolution and it is characterized by the high level of resolution in the imaging of urinary tract anatomy and the fact that it provides a lot of information about kidney functionality.^4^

Modern MRU is performed on the basis of two different imaging strategies. The first technique utilizes unenhanced, heavily T2-weigted pulse sequences to obtain static fluid images of the urinary tract. T2-weighted MR urograms have proved to be excellent in the visualization of the markedly dilated urinary tract, even if the renal excretory function is quiescent. Static-fluid MRU is less suitable for imaging of disorders that occur in the non-dilated collecting system. The second MRU technique is analogous to the methodology of the conventional intravenous pyelography and is designated as excretory MRU. For this purpose, a gadolinium chelate is intravenously administered and after its renal excretion, the gadolinium-enhanced urine is visualized using fast T1-weighted gradient echo sequences. The combination of gadolinium and low dose furosemid is the key for achieving a uniform distribution of the contrast material inside the entire urinary tract. Gadolinium excretory MRU allows to obtain high quality images of both non-dilated and obstructed urinary tracts in patients with a normal or moderately impaired renal function.^5^

After the application of contrast medium the signal changes. This is correlated with the perfusion, concentration and excretion of contrast. These changes can be analyzed sequentially in the cortex and the medulla. The anatomy and the morphological characteristics of the urinary tract are best analyzed in T2 time and after the application of contrast medium. The information about the urinary tract function comprises of transit time circulation, signal intensity curves compared to a time curve and different estimations of renal functionality.^6^

The introduction of MRU in order to obtain anatomical and functional information gives us the new opportunity to establish the pathophysiology of the urinary tract.^1^,^7^

### Imaging Technique

MRU protocol consists of conventional T1, fast spin echo (FSE) T2-weighted sequences prior and after the administration and 3D gradient echo sequences after the contrast administration.^1^,^2^,^8^,^9^

The important part of the protocol is the forced hydration of all the patients by an intravenous infusion of lactated Ringer’s solution (10 ml/kg). All the children younger than seven years of age, require sedation prior to the examination.^1^ Once the scout images, which must contain both the kidneys and bladder, are acquired the axial T2-weighted (TR-5,600; TE-160, ETL-23) images through the kidney are obtained.

Furosemide is administered intravenously (1 mg/kg up to maximal dose of 20 mg).^1^ In children the furosemide is almost always administered 15 min before contrast. This practice is supported by three reasons: a) the urinary tract is distended, b) the gadolinium concentration is diluted this reduces the susceptibility of artefacts and helps to maintain the signal change which is registered and c) the examination time is shortened which is very important in paediatric population since in spite of sedation the children are not completely calm.^1^,^9^ Then, coronal 2D, T1-weighted (TR=475, TE=17), T2-weighted (TR=5.500, TE=210, ETL=29), T2-weighted 3D (TE=600, ETL=109) sequences are acquired.

2D series are useful for the study of anatomical details, while the heavily T2-weighted 3D scans are used to create the pre-contrast maximum intensity projection (MIP) of the collecting system, ureters and bladder. Ad dynamical acquisition starts approximately 15 minutes after the administration of furosemide which coincides with the administration of contrast in the dose of 0.1 mmol/kg Gd-DTPA with the remark that the contrast injection lasts for 40 seconds. Dynamic series are acquired until both ureters are clearly visualized and for each volume acquisition MIP is automatically generated. After the completion of dynamical series high spatial resolution 3D images are acquired. These 3D images are very useful and valuable in the evaluation of the anatomical malformations including ureteric strictures, ectopic ureteric insertion as well as the postoperative appearance of the urinary tract.^1^,^9^

In cases where there are no serious obstruction of collecting system total imaging time of MRU is 45 minutes, but in cases of the severe disorder of the excretory function the imaging time can be even 1 hour. The delayed high-resolution anatomic images are particularly valuable in the evaluation of congenital malformations including ureteric strictures, ectopic ureteric insertion as well as the complex postoperative anatomy.^1^

### Sedation and preparation for examination

In most cases the sedation is necessary for the children younger than seven years of age. The children younger than two years are sedated by the administration of chloral-hydrate per-rectum, whereas older children before the MRU undergo a pediatric examination and after that the intravenous sedation is administered. For the underage patients parents must consent to their child undergoing the MR examination.

## Patients and methods

During the period of one year (January 2010 – December 2010) we included in the study 21 patients on which we performed MRU. All of our patients were in different phases of the clinical examination: patients with treatment dilemma – conservative or surgical, operated patients, patients with new diagnosed congenital malformations and patients who needed the follow up of therapy effects.

We included in this study the children of different ages: the youngest patient was 2 months old and the oldest patient was 17 years old, the average age of patients was 7.05 years. Of all the patients 43% (n=9) were females and 57% (n=12) were males.

Ultrasound as a screening method was previously performed on all the patients and in some cases other diagnostic procedures (intravenous urography, voiding cystouretrography). All of the patients positively diagnosed with hydronephrosis (n=14) had undergone intravenous urography which was insufficient to establish the cause of the above named condition and were therefore recommended for further investigations via MRU. Where hydronephrosis was suspected (n=6) to have been caused by vesicoureteral reflux (VUR), voiding cystouretrography was also conducted as well as intravenous urography. VUR was positively established as a cause in 3 patients but due to non-typical appearance of hydronephrosis MRU was conducted to eliminate other eventual anomalies which may have remained unidentified via other methodologies. In the case of remaining 3 patients VUR was not established as a cause and due to a diagnostic dilemma patients underwent MRU.

In five patients where single or multiple cysts were identified via ultrasound, MRU was conducted to analyse the form and the shape of the cysts and their potential compressive effect on the collecting system. In the case of a patient with ultrasonographically suspected malign renal tumour with compression on collecting system the second used method was MRU because of its capability to permit the simultaneous morphological and functional analysis, while other methods were avoided in order to reduce the ionizing radiation. With one patient with repeated urinary infection and regular ultrasound finding the clinician requested MRU and the classical examination methodology were avoided.

MRU is the type of examination that requires the preparation because the procedure alone lasts for a quite long period of time. The preparation and the examination together are approximately 1 hour and 45 minutes long. In the case of younger patients the sedation was necessary, all other patients were explained that the examination lasts for a quite long period of time and that during the examination they must remain still and lie calm.

All the examinations were performed on 1.5 T machines (Avanto, Siemens, Erlagen, Germany) and 3.0 T machines (Trio Tim, Siemens, Erlangen, Germany).

## Results

**FIGURE 1:**
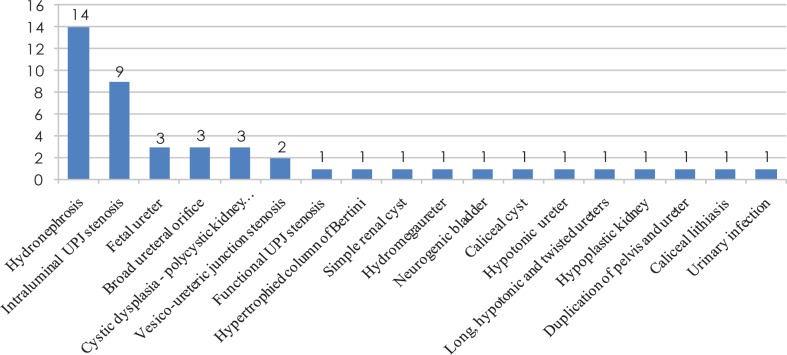
MR urography findings.

All 21 MRU examinations were successfully completed. MR urography images were diagnostically sufficient in all patients. A total of 42 kidneys, 42 collecting systems and 21 bladders in 21 patients were examined. The results of MR urography are presented in Figure 1.

In this study the most common indication for MRU was hydronephrosis of unclear aetiology: 66.7% (n=14) of all our patients were admitted with this diagnosis. In the case of 4 patients where it was challenging to establish the cause of hydronephrosis via classical methods, MRU solved a diagnostic dilemma and established that hydronephrosis was caused by intraluminal ureteropelvic junction (UPJ) stenosis.

MRU was performed on 19% (n=4) of patients with a hydronephrotic collecting system as a part of the postoperative evaluation after pyeloplasty. MRU identified the postoperative presence of functional UPJ stenosis as a cause of hydronephrosis in 1 patient with the additional finding of UPJ stenosis on the contralateral side which wasn’t identified with other methods, while in the remaining 3 patients MRU demonstrated that hydronephrosis was the continuation of previously treated stenosis and in one case the additional finding was caliceal lithiasis.

In one case of unclear hydronephrosis the cause was both UPJ and vesico-ureteric junction (VUJ) stenosis with hydromegaureter and the additional finding was UPJ stenosis on the contralateral side.

In two cases of hydronephrosis MRU established not only the cause – fetal ureter, but gave additional findings: UPJ stenosis on the contralateral side in one case and VUJ stenosis on the contralateral side in the second case.

With two patients, where based on previously conducted methodologies (intravenous urography and voiding cystouretrography), hydronephrosis was proved to be connected to VUR, MRU not only identified established insufficient broad ureter orifice but also provided further information. In one case, MRU established the occurrence of neurogenic bladder and hypotonic ureter with further signs of the urinary infection. In the case of the other patient MRU established the condition of urine retention with UPJ stenosis and fetal ureter on the contralateral side.

With one patient where renal hypoplasia and hydronephrosis of the kidney caused by VUR were diagnosed via classical methodology, this diagnosis was corroborated by MRU results which further established the condition of duplication of pelvis and ureter on the contralateral side.

**FIGURE 2:**
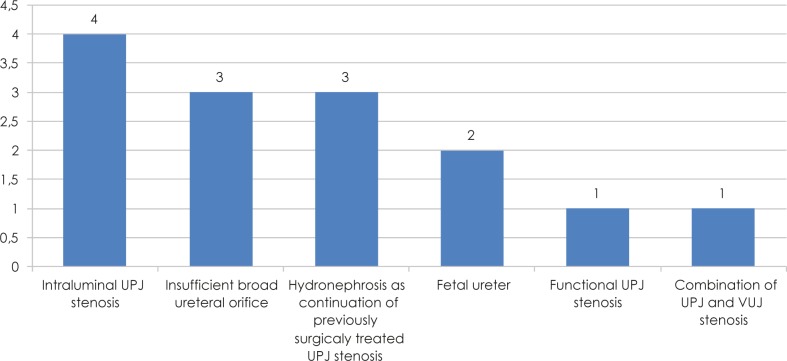
Cause of hydronephrosis.

Figure 2 contains the MRU identified pathological conditions that caused hydronephrosis.

MRU identified 6 stenosis at the level of UPJ associated with the hydronephrosis: 5 of this were intraluminal and 1 was functional.

In two patients where cysts were identified *via* ultrasound, MRU not only enabled the diagnosis of cyst type but also the analysis of cyst influence on the collecting system: in one of these two patients a caliceal cyst was identified establishing no repercussion on the collecting system, while in the other patient a simple cortical cyst with a subtle compressive effect on the upper and middle region calices was diagnosed.

MRU conducted on 3 patients, where previously polycystic disease of the kidney - cystic dysplasia was diagnosed via ultrasound, enabled the morphological analysis of cysts, renal parenchyma and collecting system and the analysis of functional capabilities of kidney which were normal in all three cases.

With one patient who previously had normal ultrasound results but had repeated urinary infection MRU revealed long, hypotonic and twisted ureters.

**FIGURE 3:**
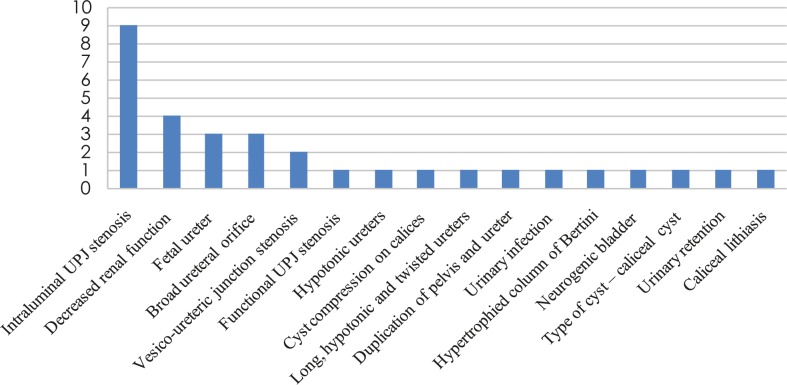
Additional MR urography findings.

Before performing MRU, 100% (n=21) of our patients were examined with one or more other diagnostic modalities (US, intravenous urography, voiding cystouretrography). MRU findings gave additional information about existing pathological conditions in 100% (n=21) of patients, which have not been identified with other mentioned modalities. In Figure 3 additionally acquired information after performing MR urography is presented.

**FIGURE 4:**
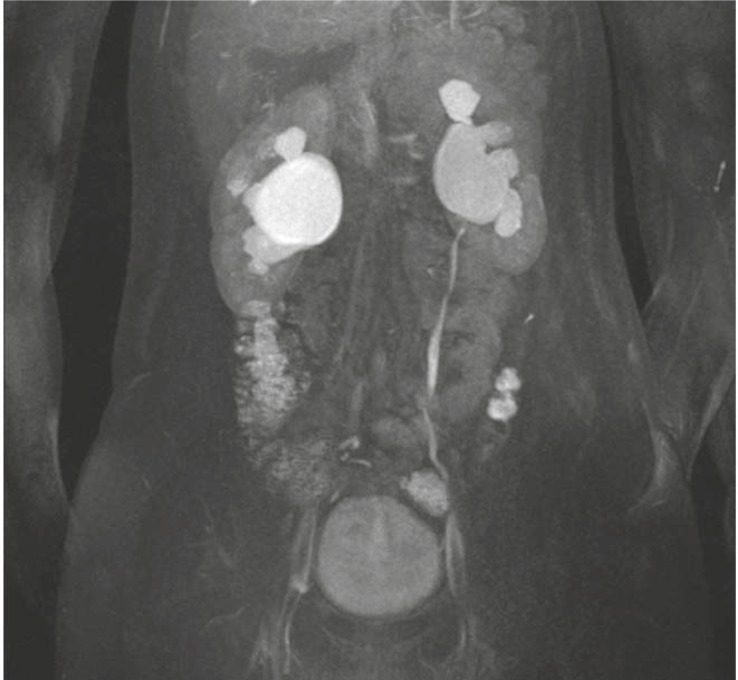
Intraluminal UPJ stenosis on the right causing hydronephrosis. Hydronephrosis on the left side is continuation of previously treated stenosis.

**FIGURE 5:**
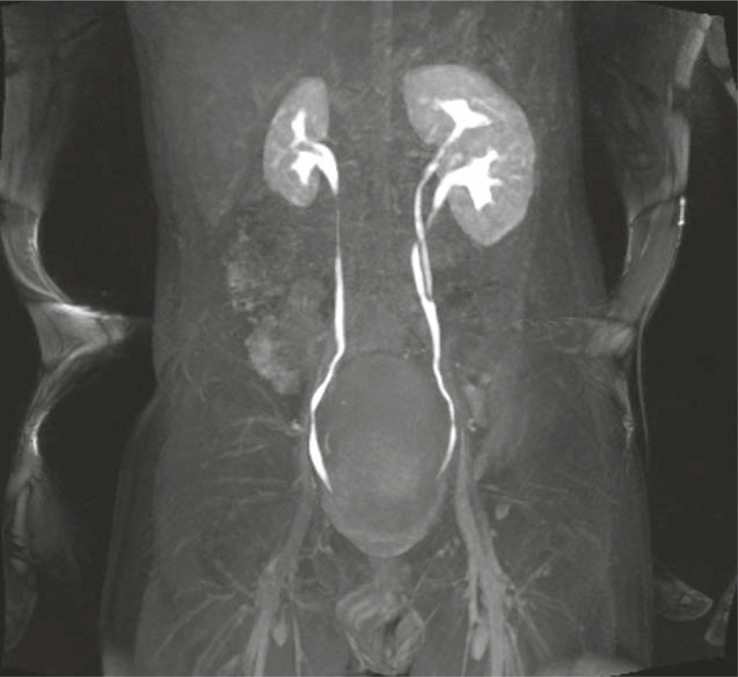
Duplication of pelvis and ureter on the left side. Hypoplastic right kidney with vesico-ureteral reflux, grade III.

**FIGURE 6:**
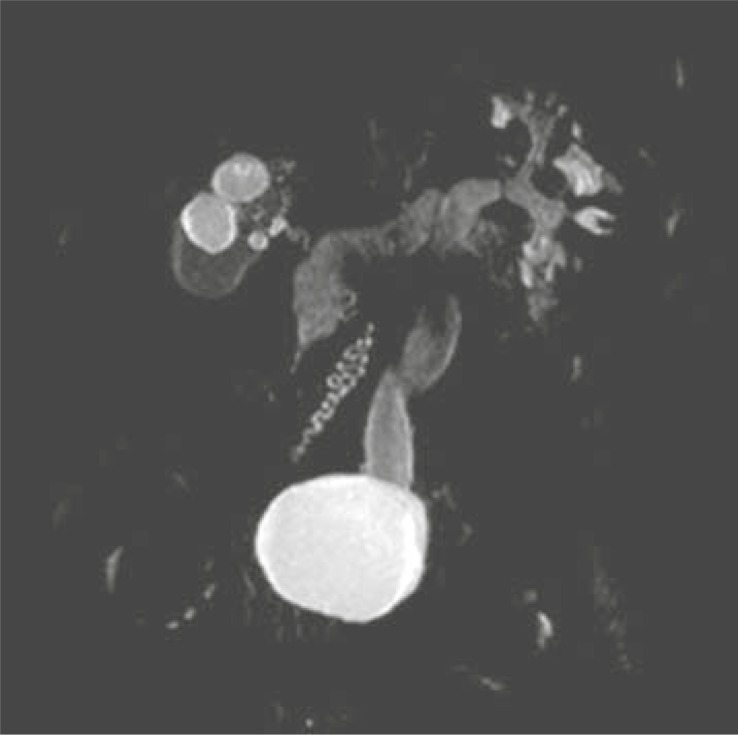
Right kidney is polycystic with fetal ureter. Hydronephrotic left collecting system with vesico-ureteric junction stenosis.

**FIGURE 7:**
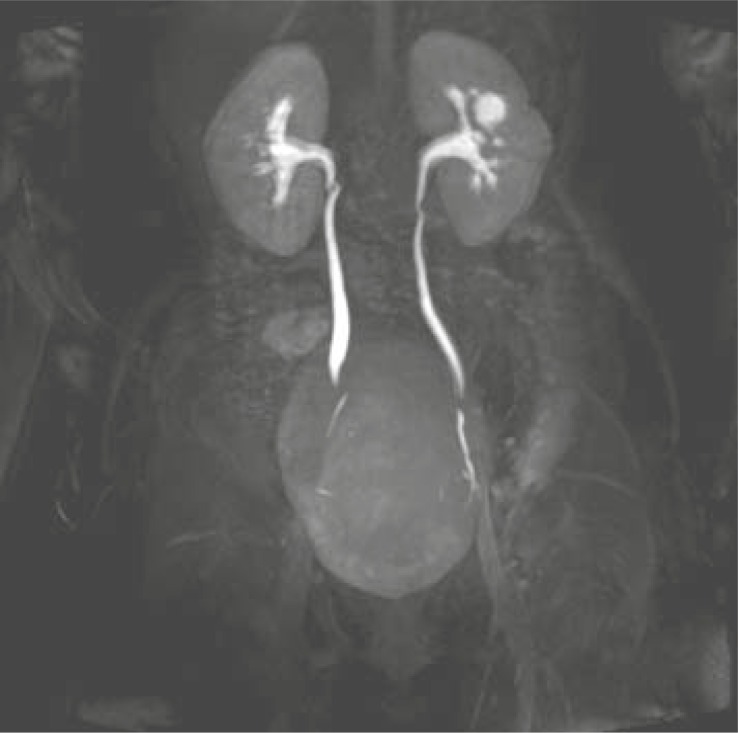
Caliceal cyst of left kidney. Vascular impression on the proximal ureter on the right side.

As a part of MRU examination we evaluated the renal function of 100% (n=21) of our patients. In 19% (n=4) of patients we identified the decreased renal function. We performed MRU due to the suspicion of malign tumour only in 4.8% (n=1) of patients. MRU finding denied the malign diagnosis: we identified the benign condition – hypertrophied column of Bertini (Figures 4–7).

## Discussion

Urinary tract diseases belong to the most frequent paediatric pathology. In the assessment of urinary tract frequently used there are traditional diagnostic procedures such as ultrasound, intravenous urography, voiding cystourethrography, CT and renal scintigraphy. Each of these procedures has some advantages and disadvantages.

Intravenous urography is known to be cost-effective but the reduced image quality by super-positioned bowel gases, prolonged examination time, the use of ionizing radiation which is mutagenic and the use of iodinated contrast media are the disadvantages.^6^,^7^,^9^,^10^

Voiding cystourethrography is considered to be “gold standard” for the diagnosis of VUR but on the other hand the risk of the urinary infection following the catheterization together with the use of ionizing radiation is relative drawbacks of this method.^11^

Ultrasound is a non invasive non ionizing method but cannot assess the renal function and analyze non-dilated ureters despite the fact that it is an operator dependent method.^11^

Renal scintigraphy uses a low radiation dose and has as a characteristic low spatial resolution and long examination time^7^,^12^; the main disadvantage of CT is a very high radiation dose and its DNA damage effect is well known.^10^,^13^

The introduction of MRU was the answer to the problems regarding the assessment of both function and morphology of the urinary tract. The advantages of this diagnostic modality are not only the non-use of ionizing radiation, but the ability to acquire high contrast and spatial resolution images in any orthogonal plane.^1^,^2^

MRU is very efficient in the assessment of different pathological conditions such as malignant renal tumours, which are very frequent among solid tumours in young children^14^, benign solid masses, renal cysts, infections, parenchymal ischemia and haemorrhage, different types of obstructions and anomalies; in addition it allows the quantification of corticomedullary perfusion and renal excretory function.^6^,^15^,^16^ The main disadvantages of MRU are: high cost, it requires sedation and hydration.^1^–^3^

The most common indication for MRU is the evaluation of hydronephrosis.^2^–^6^,^15^,^16^ The hydronephrosis in the most cases is the consequence of the obstruction of the urine flow in any point from the kidney to the bladder, and in the minor number of cases of non-obstructive uropathy.^5^,^17^ In the children the obstruction is the most communally at the level of UPJ. The most severe consequence of obstruction is the renal function deterioration. In order to prevent the loss of renal function, in the assessment of hydronephrosis it is very important to establish the type of uropathy (obstructive or non obstructive) and in order of that chose the proper therapy.^17^ MRU allows the differentiation between obstructive and non-obstructive uropathies by the calculation of renal transition time (RTT). The arterial phase of the contrast agent in the dynamic study permits the identification of crossing vessels.^18^

Considering the fact that MRU not only enables depiction of whole dilated and non dilated ureters from UPJ to ureteric insertion and their analysis from all angles *via* MIP reconstruction, but it also enables the examination of the internal outlook of ureters and of external structures which may also lead to its compression, hence ureteric anatomy and pathology are well demonstrated with MRU.^19^

The comprehensive morphological and functional analysis of all parts of urinary tract, from kidneys to bladder, gained *via* MRU, offers an opportunity to study congenital malformations of the urinary tract *in vivo* with the increased anatomic resolution.^4^–^9^,^11^–^13^,^15^–^19^

Due to enabling depiction of ureteropelvic smooth wall thickening, the renal enlargement and change in the signal intensity of renal parenchyma and perirenal fat, MRU has a significant role in the evaluation of acute pyelonephritis and renal scarring.^6^

## Conclusions

Because of the ability to acquire high contrast and spatial resolution images of the whole urinary tract in any orthogonal plane, MRU enables a precise detection and differentiation of pathological urological conditions. In particular MRU is efficient in differentiating causes of hydronephrosis – one of the most common urinary tract pathologies in pediatric population. We believe that in the future, because of its advantages, MRU will replace traditional methods in the evaluation of urinary tract pathologies in children.
